# Supplementary material for: Twist-assisted all-antiferromagnetic tunnel junction in the atomic limit
Source: Nature. 2024 Aug 14;632(8027):1045–51. doi: 10.1038/s41586-024-07818-x (PMC11358014; doi:10.1038/s41586-024-07818-x)
Supplement: Supplementary file 1 — This file contains Supplementary Figs. 1–13 and Supplementary Notes 1–3. The supplementary figures show the details of device fabrication, transport results of further MTJs based on intrinsic CrSBr layers, transport results of further twisted MTJs and further DFT results. The supplementary notes discuss pinning strength, twisted CrSBr monolayer/monolayer MTJs and the magnetization process. [file 41586_2024_7818_MOESM1_ESM.pdf]

nature portfolio

https://doi.org/10.1038/s41586-024-07818-x

Supplementary information

Twist-assisted all-antiferromagnetic tunnel junction in the atomic limit

In the format provided by the authors and unedited

Contents

Supplementary Figs. 1-13

Supplementary Notes. 1-3

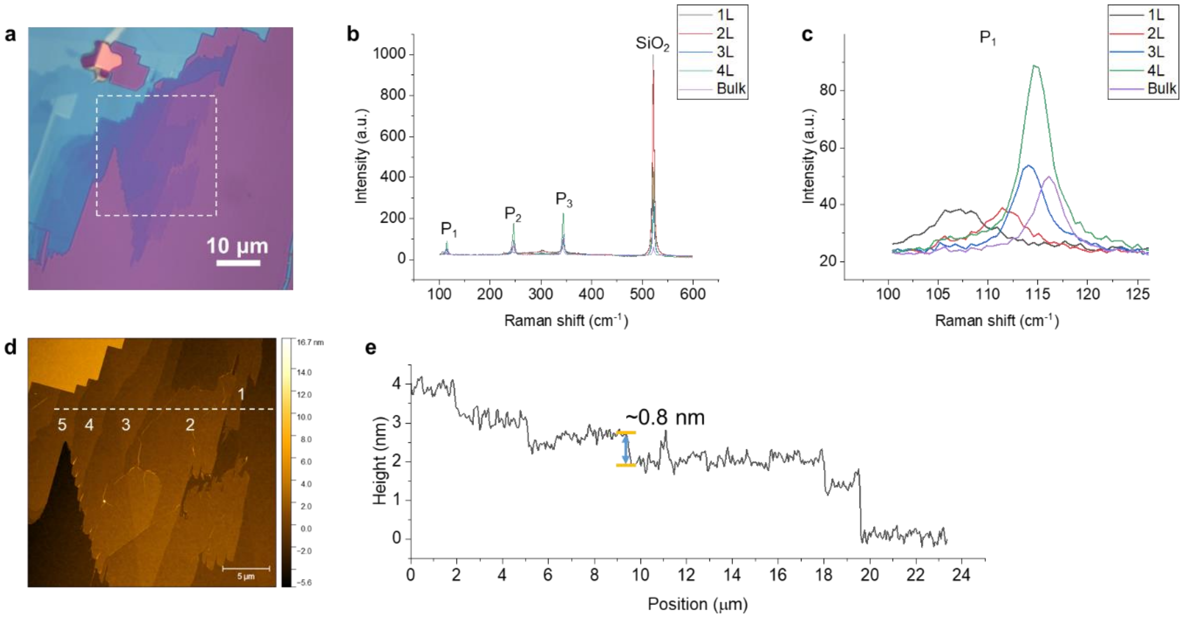

Supplementary Fig. 1 | Atomic force microscopy and Raman spectroscopy *versus* CrSBr thickness. a, Optical image of a CrSBr flake exfoliated onto Si/285 nm SiO_2. **b**, Thickness-dependent Raman spectra. **c**, Close-up of the P_1 peak in **b**. **d**, Mapping of the region defined by the white dashed line in **a** by atomic force microscopy. **e**, Thickness profile along the white dashed line in **d**, which shows ~0.8 nm steps corresponding to the thickness of a CrSBr monolayer.

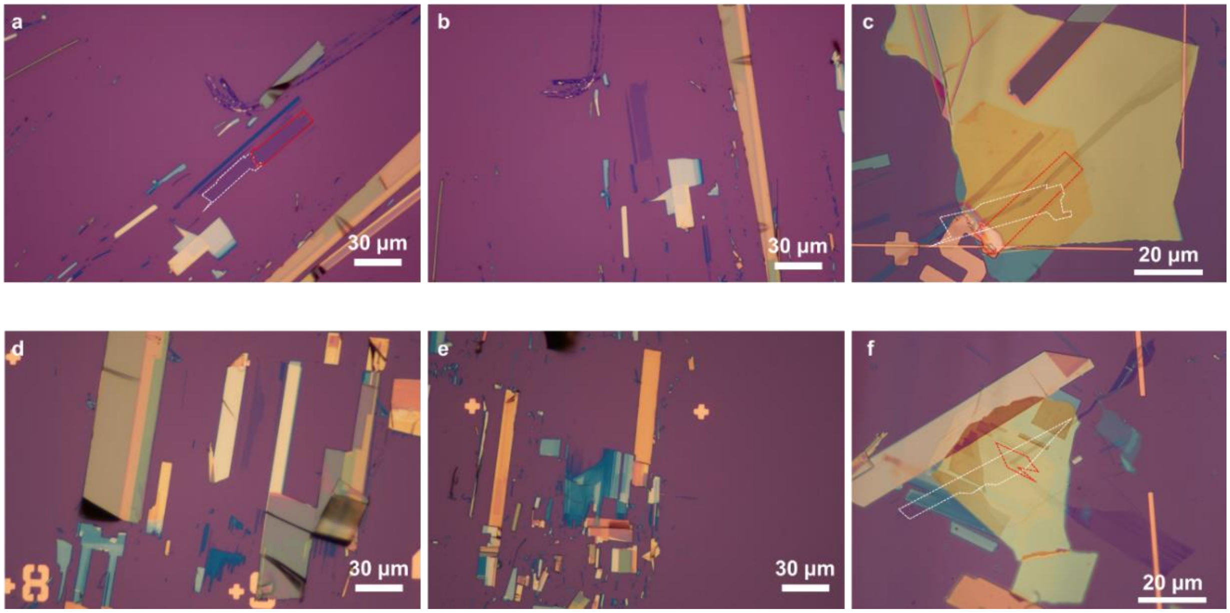

Supplementary Fig. 2 | Alignment of twisted CrSBr MTJs. a-**c**, Fabrication based on tear-and-stack method. Half of a CrSBr flake (shown by the red dashed line in **a**) was first picked up. Then this flake was twisted by a certain angle and the remaining half (shown by the white dashed line in **a**) was picked up. **d**-**f**, The angle between the two flakes was determined using the straight edge of each flake as a reference.

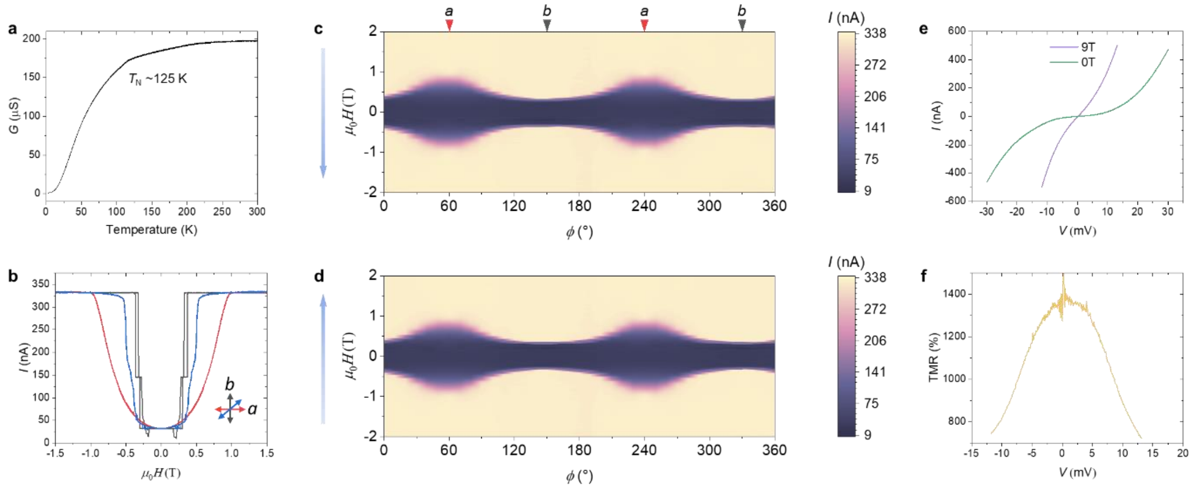

Supplementary Fig. 3 | Electrical transport results for an intrinsic 3-layer CrSBr MTJ. **a**, Conductance *versus* temperature at ZF. **b**, Tunneling current *versus* field at 2 K with field oriented along different directions as indicated by the inset. A constant DC bias of 10 mV is applied. **c**-**d**, Field orientation dependence of the tunneling current in the *ab* plane at 2 K. A constant DC bias of 10 mV is applied. The two blue arrows indicate the field sweeping direction, backward sweeping in **c** and forward sweeping in **d**. **e**, *I*-*V* curves at ZF and 9 T. **f**, Extracted TMR ratio as a function of bias based on the *I*-*V* curves in **e**.

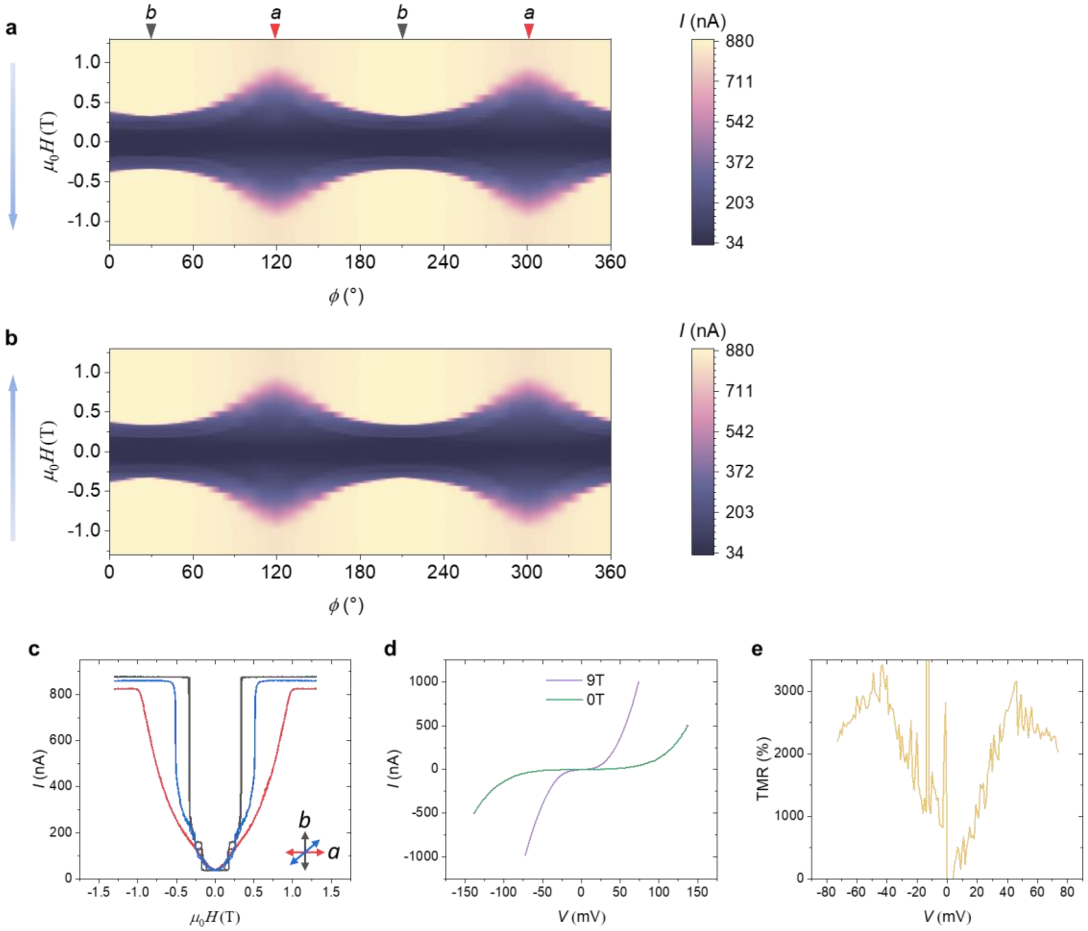

Supplementary Fig. 4 | Electrical transport results for an intrinsic 4-layer CrSBr MTJ. a-b, Field orientation dependence of the tunneling current in the *ab* plane at 2 K. A constant DC bias of 70 mV is applied. The two blue arrows indicate the field sweeping direction, backward sweeping in **a** and forward sweeping in **b**. **c**, Tunneling current *versus* field at 2 K with field oriented along the directions indicated by the inset. A constant DC bias of 70 mV is applied. **d**, *I*-*V* curves at ZF and 9 T. **e**, Extracted TMR ratio as a function of bias based on the *I*-*V* curves in **d**.

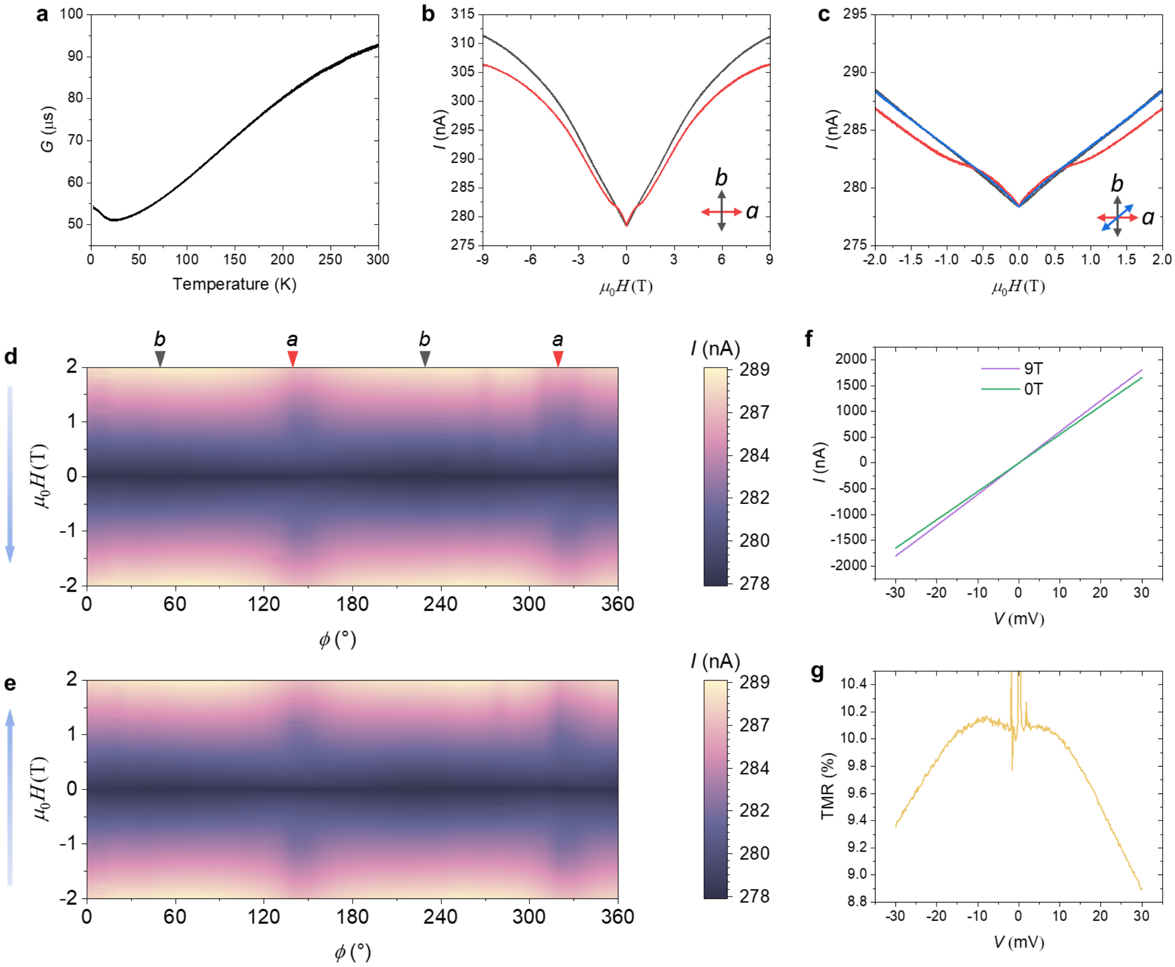

Supplementary Fig. 5 | Electrical transport results for an intrinsic monolayer CrSBr device. a, Conductance *versus* temperature at ZF. **b**-**c**, Tunneling current *versus* field at 2 K with field oriented along different directions, as indicated by the inset. A constant DC bias of 5 mV is applied. ±9 T and ±2 T fields are used in **b** and **c**, respectively. **d**-**e**, Field orientation dependence of the tunneling current in the *ab* plane at 2 K. A constant DC bias of 5 mV is applied. The two blue arrows indicate the field sweeping direction, backward sweeping in **d** and forward sweeping in **e**. **f**, *I*-*V* curves at ZF and 9 T. **g**, Extracted TMR ratio as a function of bias based on the *I*-*V* curves in **f**.

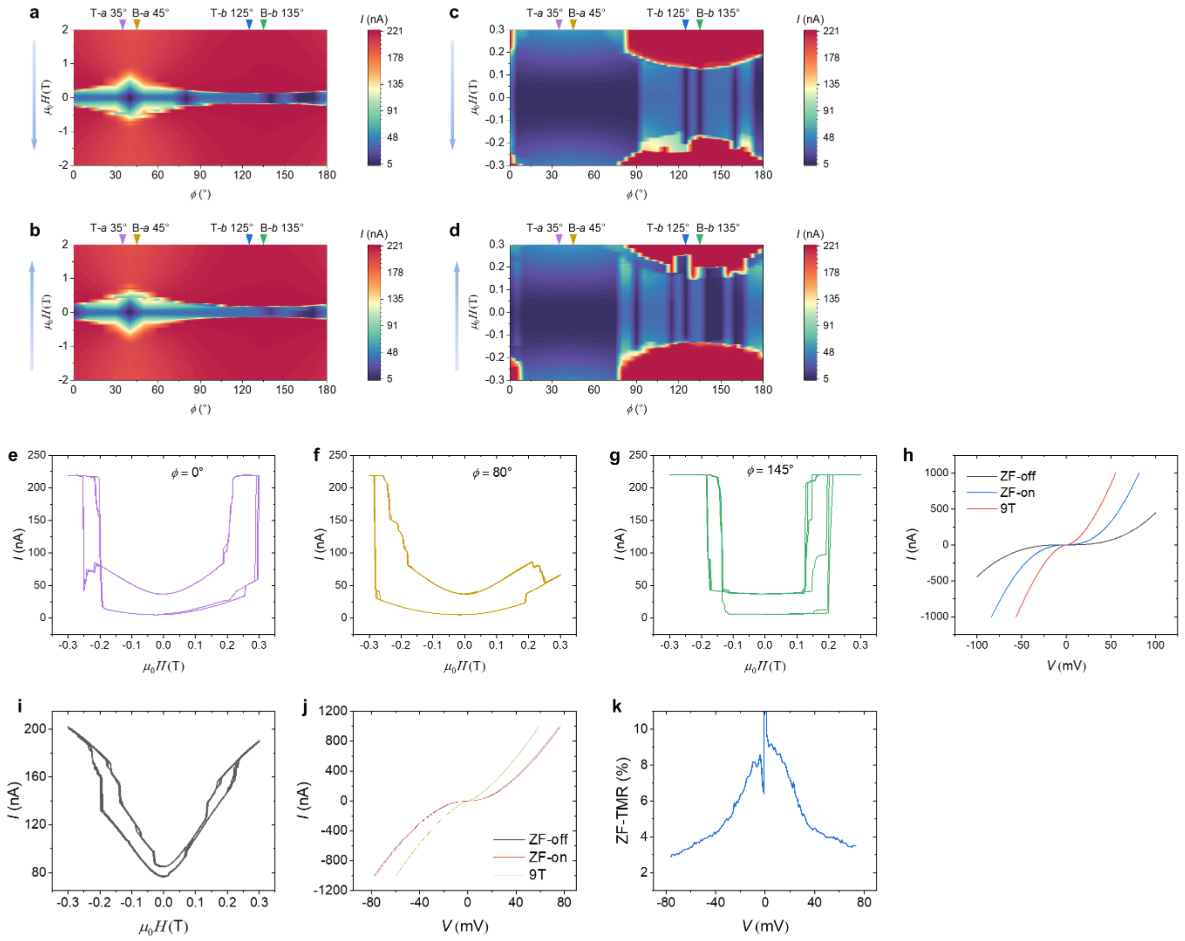

Supplementary Fig. 6 | Electrical transport results of a 10° twisted bilayer/bilayer CrSBr MTJ and an 80° twisted bilayer/bilayer CrSBr MTJ at 2 K. a-h for the 10° MTJ. **a-b**, Field orientation dependence of the tunneling current in the *ab* plane. A constant DC bias of 20 mV is applied. The two blue arrows indicate the field sweeping direction, backward sweeping in **a** and forward sweeping in **b**. **c-d**, Same as **a**, **b** except for field swept between ±0.3 T. **e-g**, Measured tunneling current for 3 repeated hysteresis loops for the field oriented at various angles. Φ = 0° in **e**, Φ = 80° in **f**, Φ = 145° in **g**. ±0.3 T field and 20 mV DC bias are used. **h**, *I-V* curves at ZF and 9 T. **i-k** for the 80° MTJ. **i**, Measuring tunneling current for 4 successive hysteresis loops for field swept between ±0.3 T. A 20 mV DC bias is used. **j**, *I-V* curves at ZF and 9 T. **k**, Extracted ZF-TMR ratio as a function of bias based on the ZF *I-V* curves in **j**.

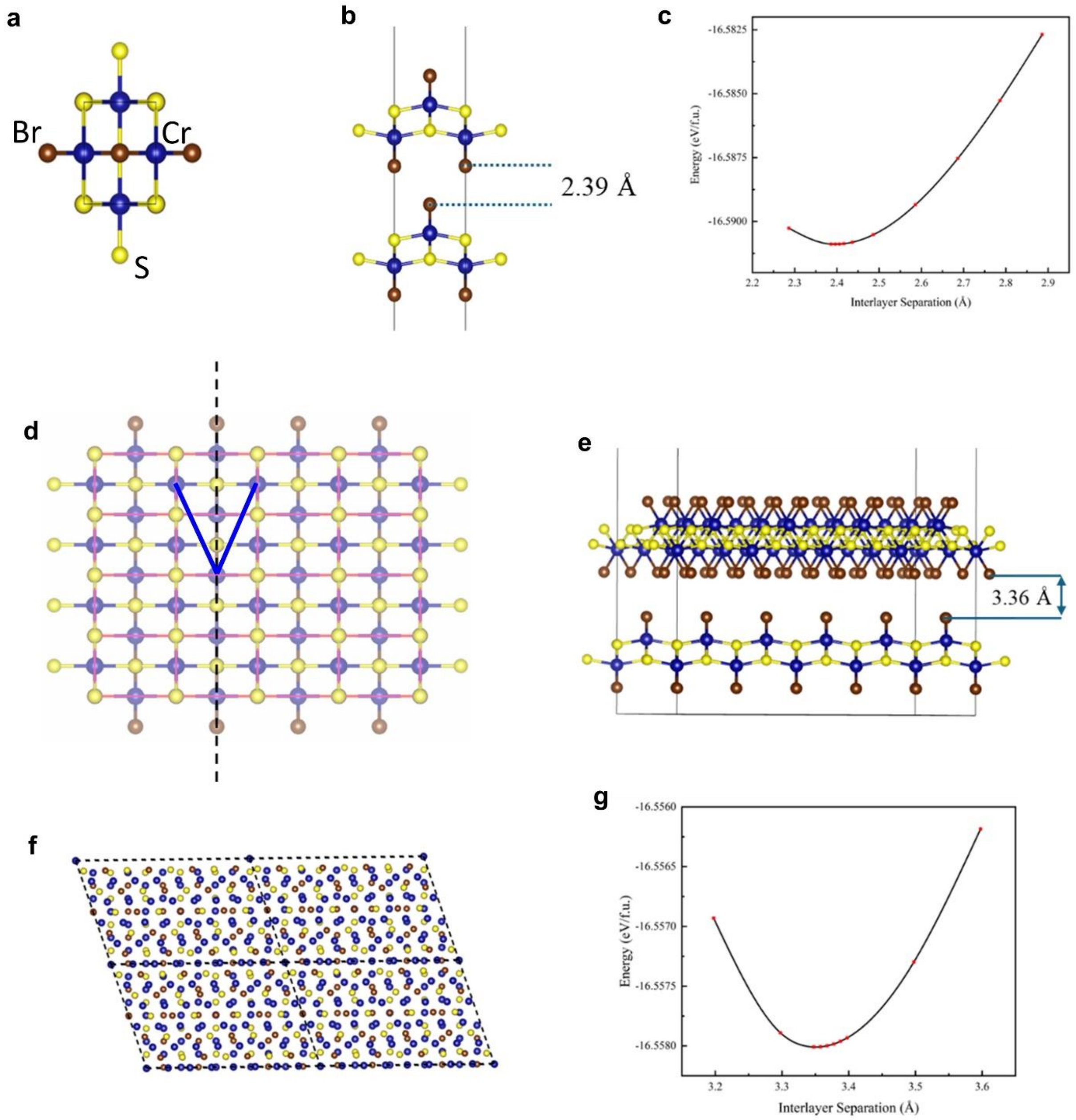

Supplementary Fig. 7 | DFT results of optimal interlayer separations. a-**b**, Top view of untwisted CrSBr bilayer (**a**). Side view of untwisted CrSBr bilayer with the optimum interlayer separation (**b**). **c**, Interlayer separation *vs* energy for untwisted CrSBr bilayer. **d**, Schematic of the choice of the twist angle for our calculations. We choose a rotation concerning the Cr atom, and we choose the rotation of the twisted layer in such a way that an atom in the (p, q) position of the untwisted layer will be below the (-p, q) atom of the twisted layer. **e**-**f**, Side view of twisted CrSBr bilayer with the optimum interlayer separation (**e**). Top view of twisted CrSBr bilayer (**f**). **g**, Interlayer separation *vs* energy graph for twisted CrSBr bilayer.

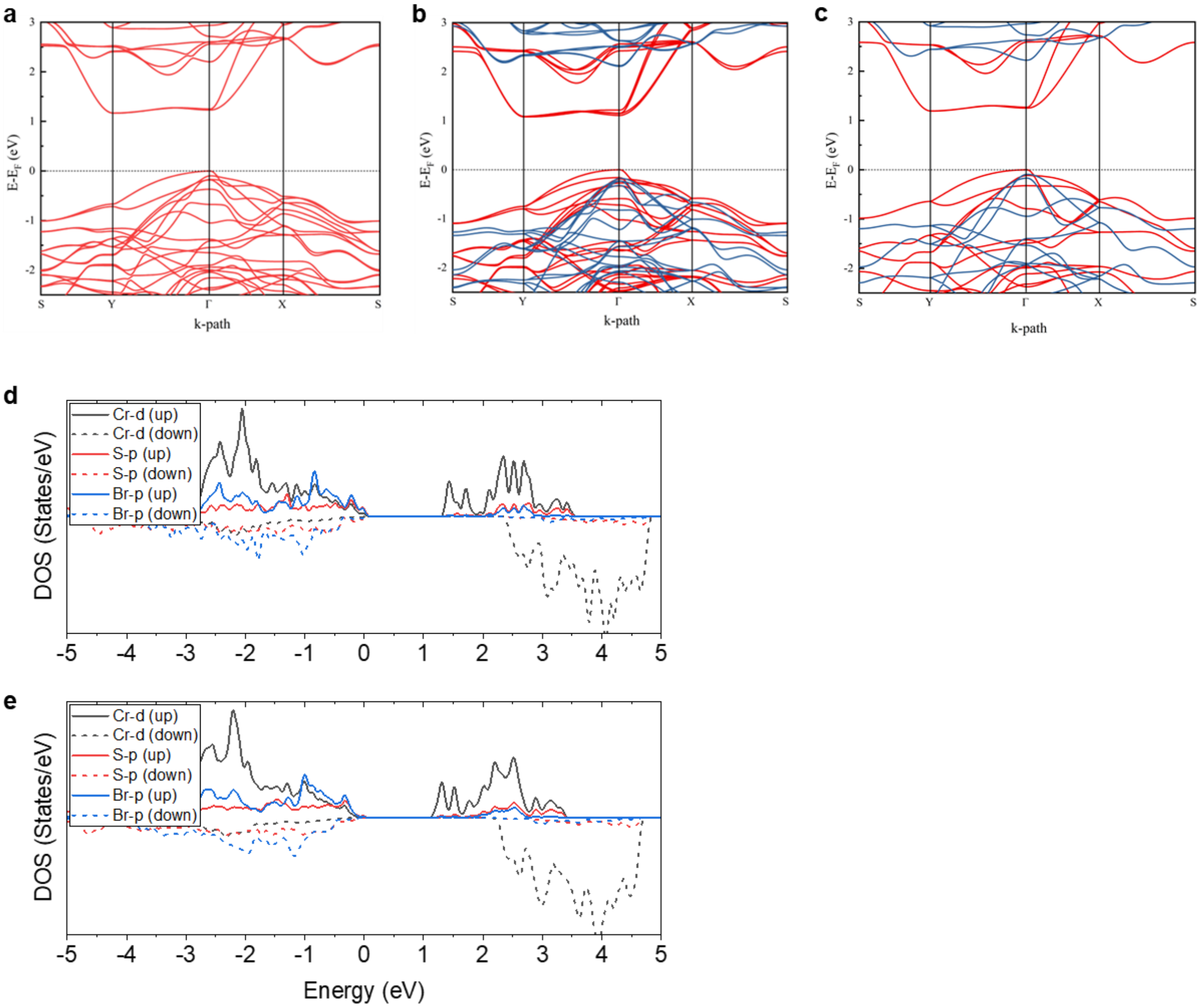

Supplementary Fig. 8 | Band structure of untwisted CrSBr bilayer and monolayer. **a**, Untwisted bilayer in antiferromagnetic configuration. The bands exhibit Kramer's degeneracy. **b**, Untwisted bilayer in ferromagnetic configuration. The red curves represent the up-spin bands, and the blue curves represent the down-spin bands. **c**, Monolayer. The red curves represent the up-spin bands, and the blue curves represent the down-spin bands. **d**, Projected DOS calculated from an individual CrSBr monolayer. **e**, Projected DOS of the top monolayer of a twisted CrSBr bilayer.

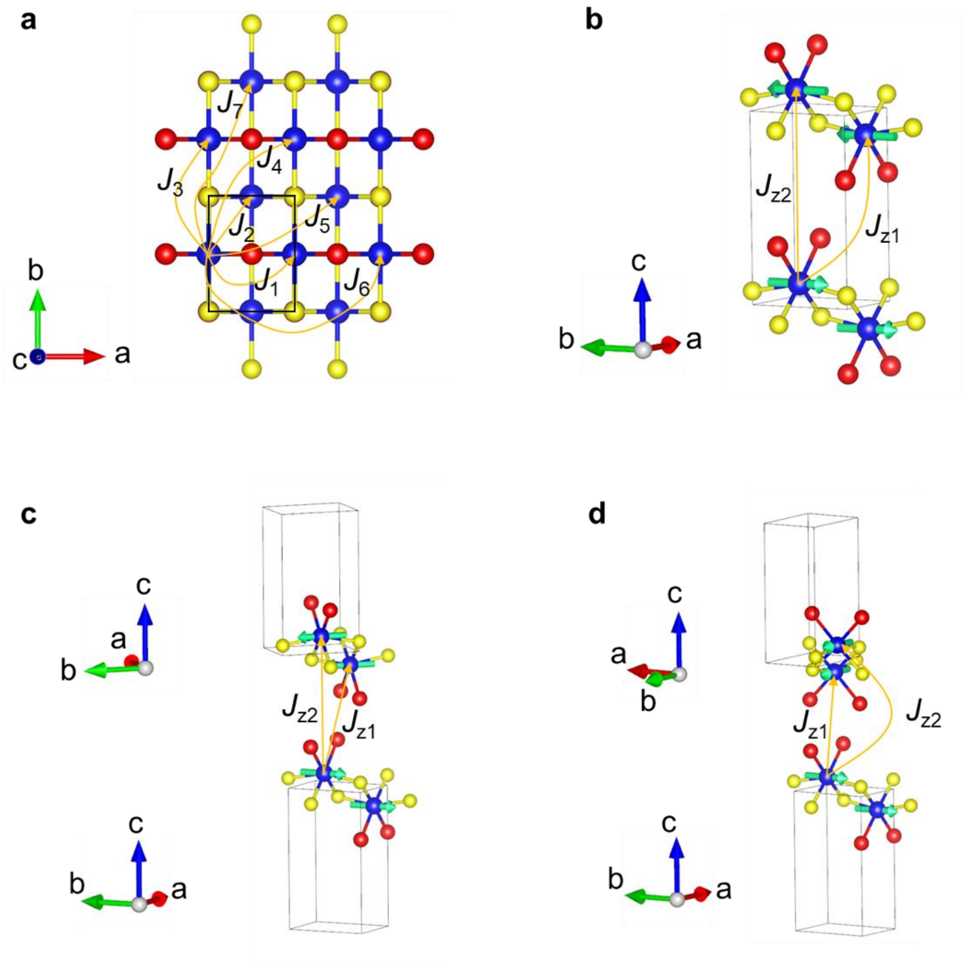

Supplementary Fig. 9 | Schematic of the magnetic exchange interactions. a, Intralayer exchange interactions of J_1 to J_7. **b**-**d**, Interlayer exchange interactions of J_{z1} and J_{z2}. Untwisted (**b**), 45°-twisted (**c**) and 90°-twisted (**d**) cases. Blue, yellow, and red balls correspond to Cr, S, and Br, respectively.

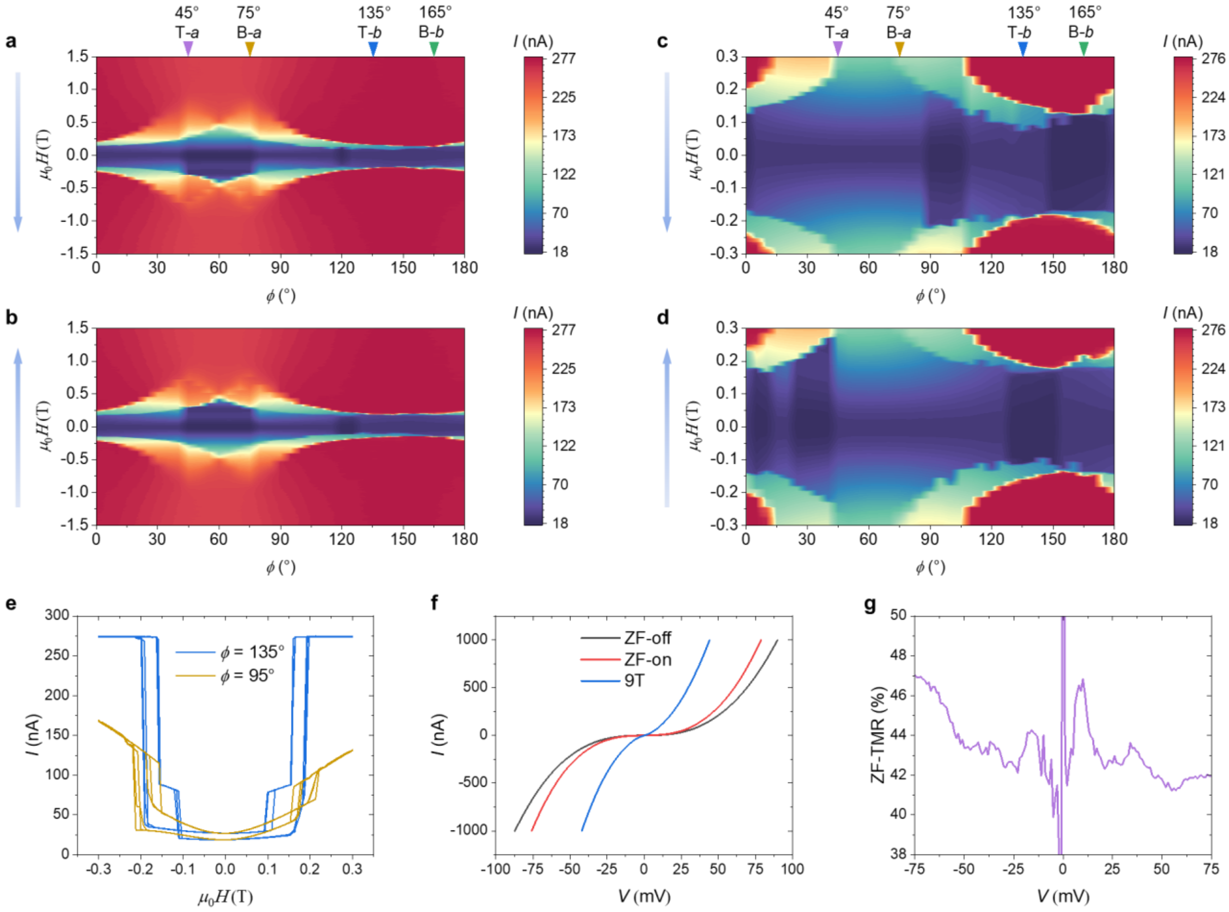

Supplementary Fig. 10 | Electrical transport results for a 30° twisted CrSBr bilayer/hBN monolayer/CrSBr bilayer MTJ. **a-b**, Field orientation dependence of the tunneling current in the *ab* plane. Two blue arrows indicate the sweeping direction of field. ±1.5 T field is used. **c-d**, Same as **a**, **b** except for sweeping field between ±0.3 T. **e**, Representatives of the two groups of ZF NV related to the easy axis (Φ = 135°) and hard axis (Φ = 95°), respectively. **f**, *I-V* curves at ZF and 9 T. **g**, Extracted ZF-TMR ratio as a function of bias based on the ZF *I-V* curves in **f**. A constant DC bias of 20 mV is applied in **a-e**.

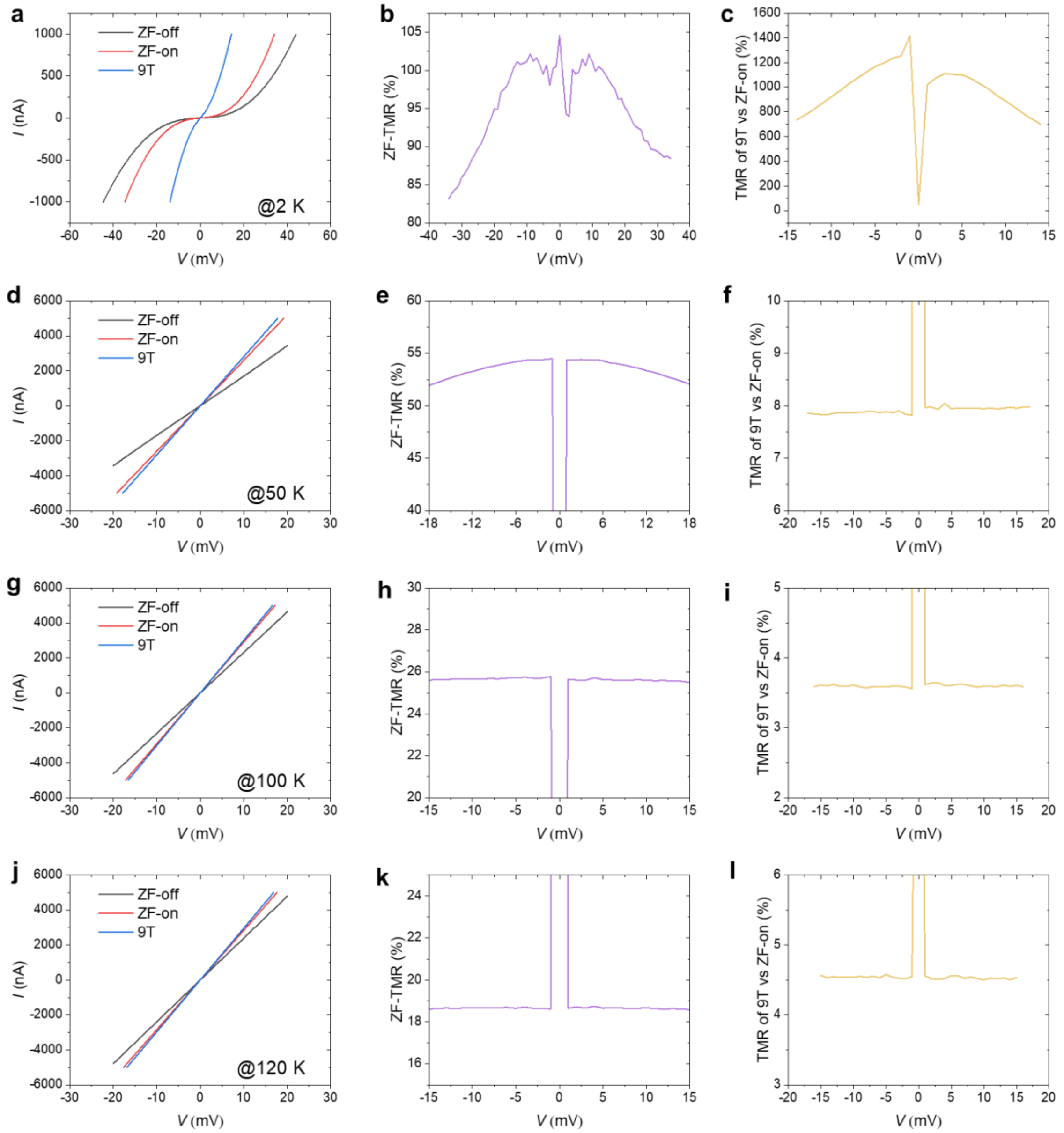

Supplementary Fig. 11 | *I-V* curves and bias-dependent TMR of a 40° twisted bilayer/bilayer CrSBr MTJ at different temperatures. The left column shows the *I-V* curves at different temperatures. The mid column shows the extracted ZF-TMR ratio as a function of bias based on the corresponding *I-V* curves of the left column. The right column shows the extracted 9 T *vs* ZF-on TMR ratio as a function of bias based on the corresponding *I-V* curves of the left column. The different temperature-dependent forms of the TMR between twisted and untwisted interfaces are independent of the applied bias.

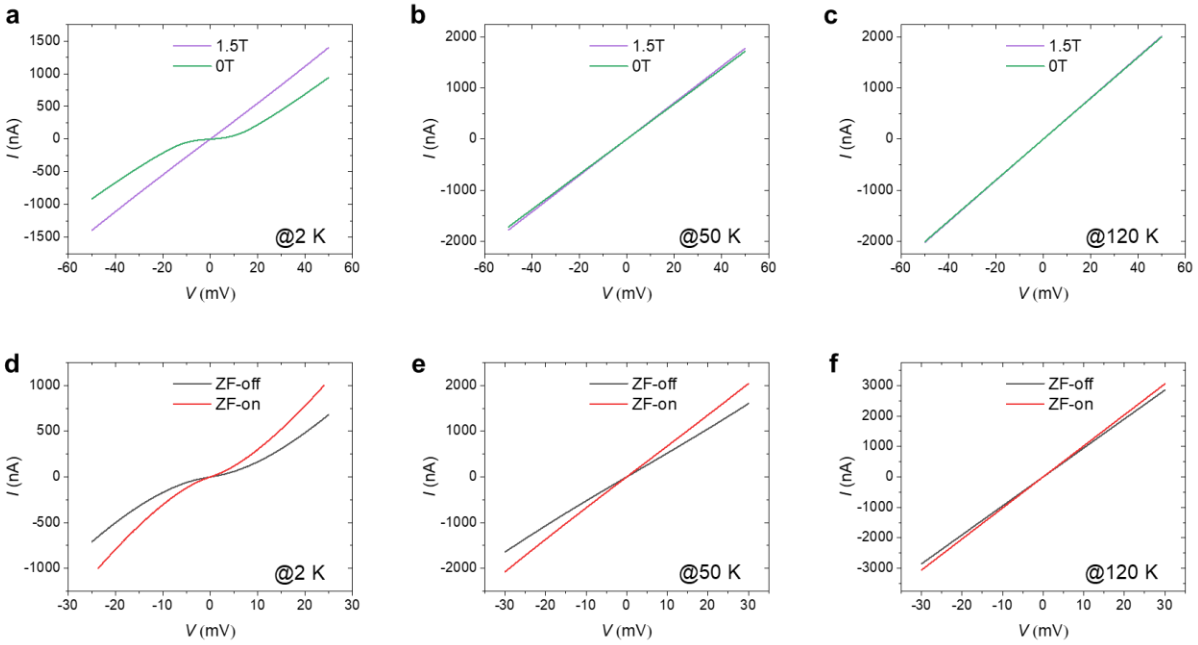

Supplementary Fig. 12 | *I*-*V* curves of an intrinsic (untwisted) bilayer CrSBr MTJ and a 45° twisted monolayer/monolayer CrSBr MTJ at different temperatures. The top panel is for the intrinsic bilayer CrSBr MTJ. The bottom is for the 45° twisted monolayer/monolayer CrSBr MTJ.

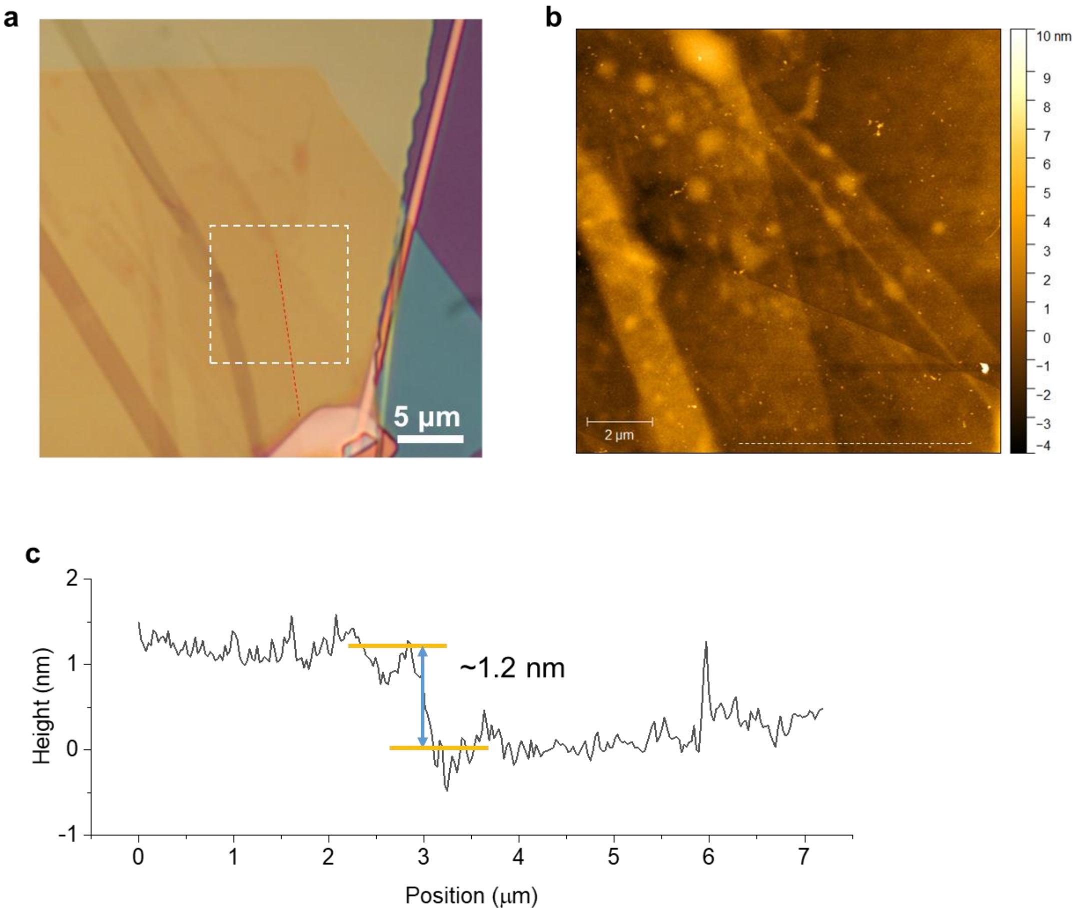

Supplementary Fig. 13 | Thicker vdW gap in twisted CrSBr bilayer. a, Optical image of a twisted bilayer MTJ. The red dashed line marks the terrace between the bottom monolayer and the overlap region of the top/bottom monolayers. **b**, Mapping of the region defined by the white dashed line in **a** by atomic force microscopy. **c**, Thickness profile along the white dashed line in **b.** The ~1.2 nm terrace is thicker than the height of ~0.8 nm in Supplementary Fig. 1e.

Supplementary Note 1

The trade-off between pinning strength and ZF-TMR

To quantify the pinning strength, we first extract the critical field (H_c) from the natural (untwisted) CrSBr bilayer's field direction-dependent tunneling currents of Fig. 2e in the main text and Extended Data Fig. 1a. When the field is oriented along *a*-axis (*b*-axis), the H_c is maximal (minimal) owing to the strong in-plane magnetic anisotropy. We further normalize H_c by dividing the maximal H_c. The grey curve in Supplementary Fig. 14a shows the α-dependent normalized H_c, in which α = 0° (90°) means the field is along the *b*-axis (*a*-axis) of a CrSBr flake (taken as the reference flake). Then, supposing there is another CrSBr flake stacked via a relative twist angle θ_{twist} on the reference flake, whose α-normalized H_c curve can be obtained by right shifting the grey curve by θ_{twist} (left shifting is also fine, which does not influence the subsequent discussion). In Supplementary Fig. 14a, the cases of θ_{twist} = 30°, 60° and 90° are plotted. Next, the discrepancy of pinning strength between the twisted double CrSBr flakes can be quantified by calculating the difference using their corresponding α-normalized H_c curves, see Supplementary Fig. 14b, for example, the purple curve of θ_{twist} = 30° in Supplementary Fig. 14b is obtained using the red curve of Supplementary Fig. 14a to subtract the grey curve of Supplementary Fig. 14a. When ΔNormalized H_c is switched between negative and positive by rotating field orientation, the pinned flake is alternated. Supplementary Fig. 14b indicates the pinning strength depends on the orientation of the external field, namely Φ in the main text.

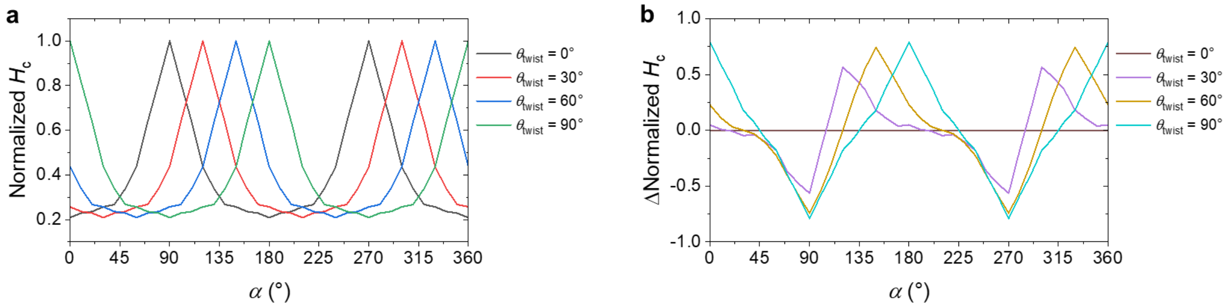

Supplementary Fig. 14 | Pinning strength. a, α-dependent normalized H_c. **b**, α-dependent Δnormalized H_c.

It is read from Supplementary Fig. 14b that a larger θ_{twist} would increase the ΔNormalized $H_{c,}$ which is favorable to observing ZF NV because of the increased pinning strength. For example, θ_{twist} = 90° can result in the maximal ΔNormalized H_c when the field is along the *a*-axis of one flake but instead along the *b*-axis

of the other flake. In contrast, if θ_{twist} = 0°, pinning vanishes (brown line of Supplementary Fig. 14b). Thus, pinning is also θ_{twist}-dependent.

Nevertheless, a larger θ_{twist} reduces ZF-TMR. For example, in our control experiment of twisted 90° bilayer/bilayer MTJ (Extended Data Fig. 5), we cannot observe any ZF NV. The relative angle θ between the two magnetizations has two values of θ_{twist} and π - θ_{twist} at ZF (see Fig. 1f of the main text), but they are equal when θ_{twist} = 90°. Moreover, the DFT results of Fig. 4e in the main text show nice consistency with the experimental data and a lower TMR with a larger twist angle. Based on the above discussion, it is confirmed that there is a trade-off between pinning strength and ZF-TMR mediated by θ_{twist}, which provide a useful knob for designing different twisted MTJs with varying twist angles according to practical applications.

We further highlight the pinning effect in the twisted MTJs compared to traditional ways. Some sort of pinning effect is one of the necessary preconditions for an MTJ to work functionally. If there is no pinning, sweeping external magnetic field would produce the same influence on the magnetizations of both magnetic layers and could randomly reach one of the bistable states at ZF. For example, applying field along the angular bisectors of the two magnetizations results in ΔNormalized H_c = 0 (Supplementary Fig. 14b). In that case, ZF NV becomes random, see experimental results in Extended Data Fig. 2j. Thus, including memory components, our twisted MTJs are also potentially used on random electronics, e.g., stochastic MTJs, and true random number generators. Moreover, the pinning strength in the twisted MTJs is twisted angle-dependent and field orientation-dependent. These exotic properties are superior to the conventional MTJs, in which the pinning effects are usually from exchange bias produced by an additional AFM pinning layer (Fig. 1b,c in the main text). In contrast, the twisted MTJs inherently possess a pinning effect arising from the strong magnetic anisotropy of CrSBr without needing an additional AFM pinning layer.

Supplementary Note 2

Additional discussion on twisted CrSBr monolayer/monolayer MTJs

The ZF NV is expected to be maintained when most of the top and bottom monolayers in Fig. 1f of the main text are removed because the twisted interface remains. We fabricated a 55° twisted monolayer/monolayer device, as shown in Supplementary Fig. 15a. Its conductance-temperature results at ZF show no evidence for a PM-to-AF transition (Extended Data Fig. 6b), which is further confirmed by the observation of a quasi-parallel spin configuration at ZF and continuing to increase the applied field, a quasi-antiparallel configuration and a new quasi-parallel configuration emerge (Supplementary Fig. 15c). ZF NV does not appear when applying a large field sweeping (Supplementary Fig. 15d). To realize ZF NV, we reduce the field sweep range, only allowing the parallel-antiparallel part of the evolution to happen. Extended Data Fig. 6c shows that ZF NV is observed along each *a-axis* of both CrSBr flakes by alternatively pinning the top and the bottom monolayers, as shown by the exemplary plots in Extended Data Fig. 6d. Moreover, these are very robust, as confirmed by the 10 successive loops that are shown in Supplementary Fig. 15e,f. Note that, in Supplementary Fig. 15e,f, the relationship of tunnel currents and external field manifesting slopes instead of plateaus in conventional FM MTJs (see the reason in Supplementary Note 3)[1,2]. Comparing the Φ = 210° results in Extended Data Fig. 6d with Supplementary Fig. 15e, the mirrored symmetry after a large field stimulation again supports the decoupled mechanism at the twisted interface. However, a ±0.3 T field is still too strong to create ZF NV along the *b-axis* of the CrSBr monolayer as distinct from the observation of the two groups of ZF NV in the twisted bilayer/bilayer MTJs. The reason is that the intrinsic AF interfaces in the twisted bilayer/bilayer MTJs, i.e., the top and bottom untwisted interfaces within the bilayers in Fig. 1f of the main text, despite not contributing ZF NV, provide a large pinning strength in the pinned bilayer due to the AF exchange interaction. By contrast, there are no such interfaces in the twisted monolayer/monolayer device, so a ±0.3 T field oriented along the *b-axis* of one monolayer flips the spins in both monolayers. If further decreasing the field to a few hundred Oersted, ZF NV along *b-axis* is also observed (Supplementary Fig. 15g). The *I-V* results are presented in Extended Data Fig. 6e and Supplementary Fig. 15h, in which the 9 T result almost overlaps the ZF-on curves also due to the absence of such untwisted interfaces. In contrast, in Fig. 3e of the main text, the 9 T *I-V* curve is remarkable because all antiparallel spin arrangements become parallel at all interfaces. The ZF-TMR ratio is more than 70% (inset of Extended Data Fig. 6e). Similar results were reproduced in another 45° twisted monolayer/monolayer device, whose ZF-TMR ratio is more than 130% owing to the smaller twist angle, see Supplementary Fig. 16. The ZF NV is still observed when inserting an hBN layer between two twisted CrSBr monolayers (Extended Data Fig. 7). The ZF-TMR of the twisted monolayer/monolayer MTJs is still robust with increasing temperature (pink curve of Extended Data Fig. 6f). We also measured the

temperature-dependent TMR of the untwisted CrSBr bilayer MTJ, which quickly decays with increasing temperature (grey curve of Extended Data Fig. 6f, note the TMR is volatile and is measured at ZF and 1.5 T). These results are in good agreement with the observations in the twisted bilayer/bilayer MTJs.

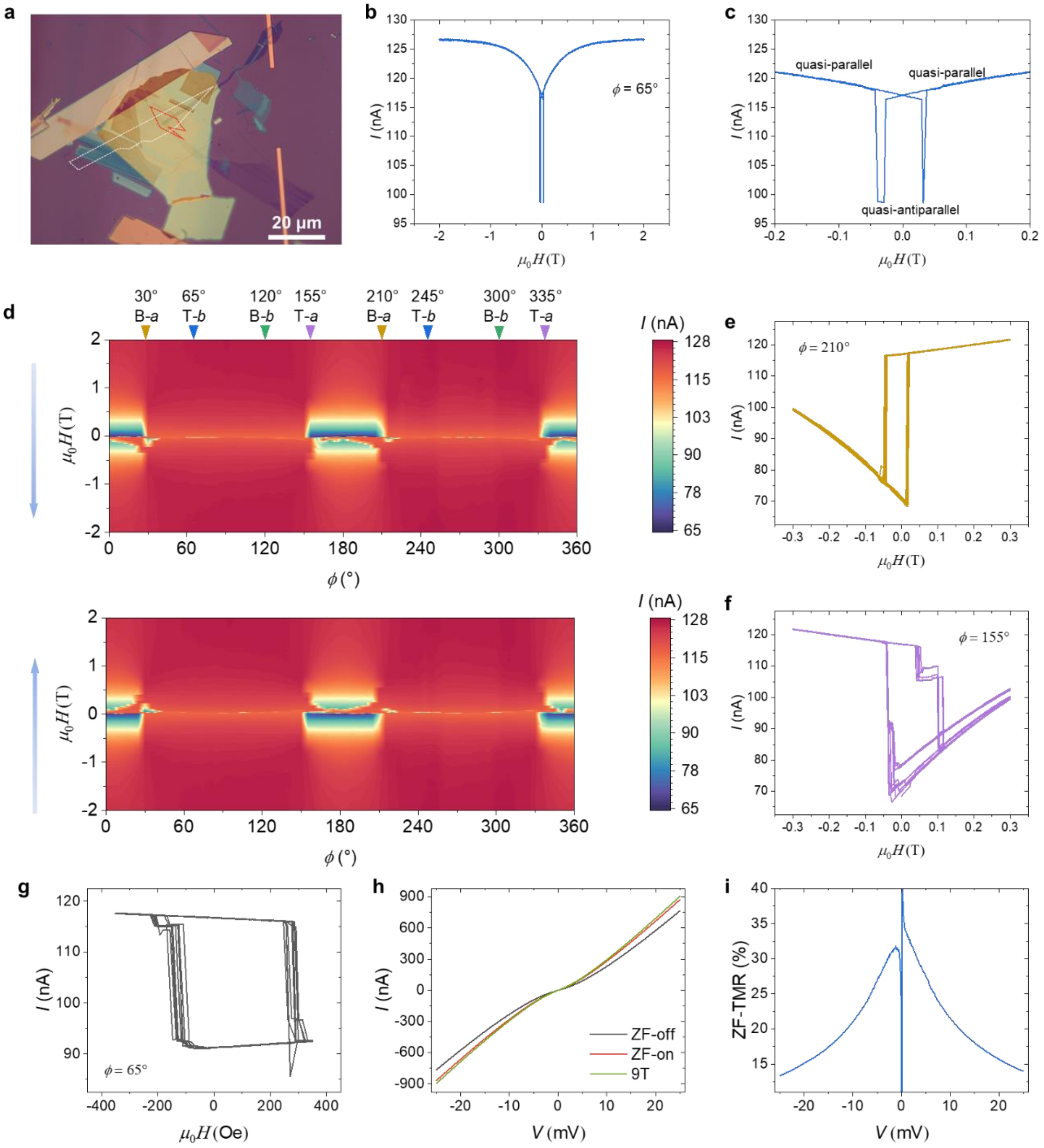

Supplementary Fig. 15 | Additional electrical transport results of the 55° twisted monolayer/monolayer CrSBr MTJ. In this figure, 5 mV DC bias is used for all related measurements except as otherwise noted. **a**, Optical microscope image of the device. The two closed shapes in red and white dashed curves outline the bottom and top flakes, respectively. **b**. Tunneling current *versus* field at 2 K with field oriented along the easy axis of the top flake. **c**, A close-up of **c** near the ZF, showing a quasi-parallel spin configuration at ZF. As the field is increased, a quasi-antiparallel configuration and a new quasi-antiparallel configuration emerge sequentially. **d**, Field orientation

dependence of the tunneling current in the *ab* plane. The two blue arrows indicate the field sweeping direction. ±2 T field is used. **e**-**f**, 10 loops for field oriented along Φ = 210° and Φ = 155°, respectively. ±0.3 T field is used. **g**, 10 loops for field oriented along Φ = 65°. ±350 Oe field is used. **h**, *I*-*V* curves at ZF and 9 T. **i**, Extracted ZF-TMR ratio as a function of bias based on the ZF *I*-*V* curves in **h**.

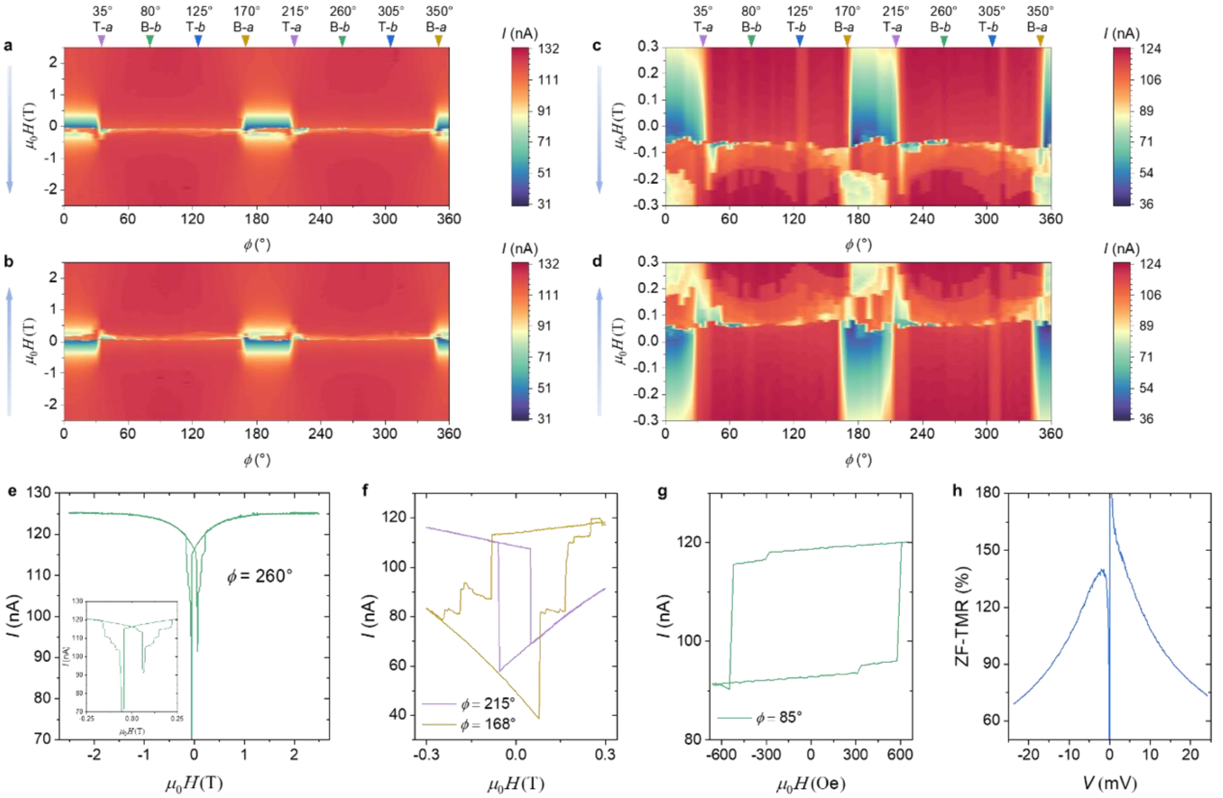

Supplementary Fig. 16 | Electrical transport results of a 45° twisted monolayer/monolayer CrSBr MTJ at 2 K. a-**b**, Field orientation dependence of the tunneling current in the *ab* plane. The two blue arrows indicate the field sweeping direction. ±2.5 T field is used. **c**-**d**, Same as **a**, **b** except for field swept between ±0.3 T. **e**, Tunneling current *versus* field for field oriented along the easy axis of the bottom flake. The inset shows a close-up near ZF. ±2.5 T field is used. **f**, Demonstration of ZF nonvolatility related to the hard axis. ±0.3 T field is used. **g**, Demonstration of ZF nonvolatility related to the easy axis. ±670 Oe field is used. A constant DC bias of 5 mV is used in **a**-**g**. **h**, ZF-TMR.

In the sweeping field experiments, we noticed multiple transitions and kinks in the colormaps of tunneling currents at finite fields, which derive from magnetic domains in the tunneling junction area since they always show randomness in multiple sweeping loops (Supplementary Fig. 17). If their origins are magnetic coupling, the multiple transitions and kinks should prefer a stable appearance instead of randomness. There are two main reasons for forming magnetic domains at finite fields. (1) Internal factors, multiple

mechanisms' competition, such as external field, magnetocrystalline anisotropy, demagnetization energy, and thermal fluctuation[3]. (2) External factors, asymmetries introduced via device fabrications, for example, local strains and defects. Comparing Supplementary Fig. 17a with Supplementary Fig. 17b, ideally, their shape should be equal regarding the symmetry of the twisted structure but rather are different, in fact, owing to such external factors.

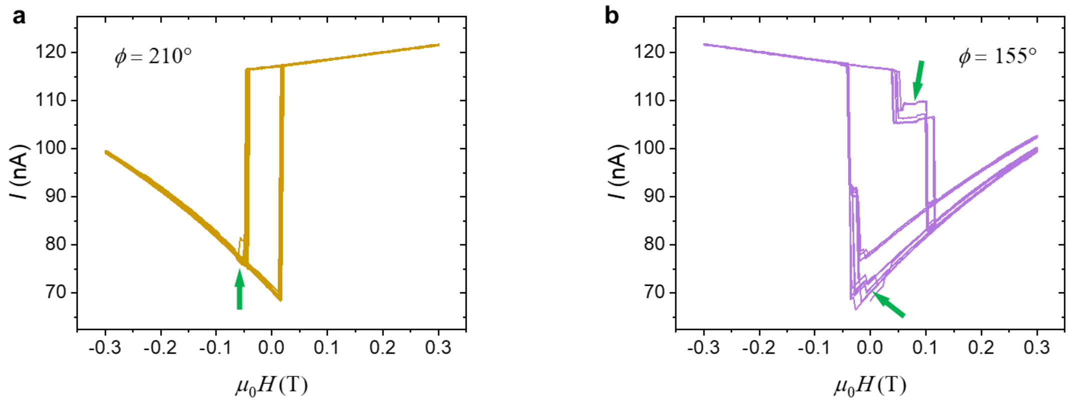

Supplementary Fig. 17 | Tiny plateaus and kinks at finite fields in multiple loops of sweeping field. a-b, The same data as Supplementary Fig. 15e,f. The arrows mark the tiny plateaus and kinks.

Supplementary Note 3

The Stoner-Wohlfarth model for the twisted CrSBr MTJs

The DFT calculations and control experiments with inserted hBN layers support the decoupled twisted interface. Based on the decoupled picture, here we examine the magnetization process using the Stoner-Wohlfarth model.

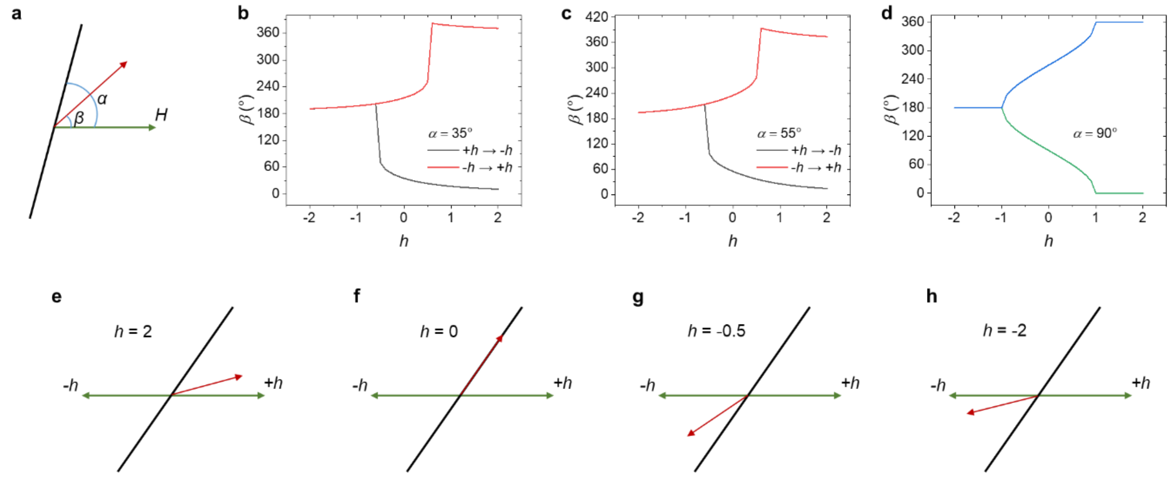

Supplementary Fig. 18 | Magnetization process of a single magnetic domain with an external magnetic field. a, A schematic diagram of magnetization (red arrow) and external magnetic field (H, green arrow). Black line is the easy axis. **b**-**d**, Magnetization processes with different α in a dual sweeping external magnetic field. **e**-**h**, Diagrams of the detailed magnetization process of α = 55°.

See Supplementary Fig. 18a, we consider a single-domain CrSBr monolayer in an in-plane magnetic field H (green arrow), which is applied at an angle α (measured counterclockwise) to the easy axis of its uniaxial anisotropy (the black line). If the magnetization (red arrow) of the CrSBr monolayer then lies at an angle of β to the magnetic field direction, the energy density (E) of the system is

$$E = K\sin^2(\alpha-\beta) - \mu_0 H M_s \cos\beta \qquad (S1)$$

The first term of the right side is the magnetic anisotropy energy, and K is the anisotropy constant. The second term is the Zeeman energy, μ_0 is vacuum permeability and M_s is the saturation magnetization. We ignore other energy sources, such as demagnetization energy, thermal fluctuation.

We further modify Eq. (S1) with a parameter $h = \frac{\mu_0 H M_s}{2K}$ to get

$$\frac{E}{2K} = \frac{\sin^2(\alpha-\beta)}{2} - h\cos\beta \qquad (S2)$$

To enhance our work's universality, we do not introduce the practical parameters of CrSBr into Eq. (S2), but instead directly calculate it. The energy can be minimized to find the direction of the magnetization at any given value of h and α.

Eq. (S2) is difficult to solve with an analytic solution at any α, but it can be solved numerically. Supplementary Fig. 18b-d shows the results of β with varying h when α = 35°, 55°, 90°, respectively. In Supplementary Fig. 18e-h, we describe how magnetization changes with sweeping h from 2 to -2 when α = 55° for an example (grey curve of Supplementary Fig. 18c). At h = 2, β = 14.28° instead of 0° because of the magnetic anisotropy energy (Supplementary Fig. 18e). Decreasing h results in the magnetization counterclockwise rotating towards the easy axis, and when h = 0, the magnetization is back to the easy axis (Supplementary Fig. 18f). Then, by increasing h along the negative direction to 0.5, the magnetization suddenly flips (Supplementary Fig. 18g). Note that β = 213.98° larger than 180° at this moment. Further increasing h forces the magnetization to rotate clockwise to approach the negative direction of h (Supplementary Fig. 18h), namely a stiffening effect. When sweeping h back to 2, an analogous process would happen as the red curve shown in Supplementary Fig. 18c. An important result in Supplementary Fig. 18c is that the magnetization shows double directions along the easy axis at h = 0, which is determined by backward or forward sweeping. The case of α = 35° has a similar magnetization process (Supplementary Fig. 18b).

Nevertheless, the case of α = 90° is different (Supplementary Fig. 18d). When h is applied along the hard axis, a strong field ($|h| \geq 1$) forces the magnetization to align to the direction of h (the magnetization is saturated). The magnetization would randomly choose a path from the green and blue curves in Supplementary Fig. 18d to evolve with decreasing h. Therefore, the magnetization will randomly reach one of the directions along the easy axis at h = 0. On the other hand, if h is not strong ($|h| < 1$), both backward and forward sweeping h always make the magnetization process evolve along the same path and deterministically reach one of the directions along the easy axis at h = 0 instead of two directions as in Supplementary Fig. 18b and c. This rule is also suitable for other cases of $\alpha \neq$ 90° when a field is not strong enough to flip spin.

Now, we consider the case of the 55° twisted CrSBr monolayer/monolayer as an example. See Supplementary Fig. 19a, the black dashed line and solid line are the easy axes of the top and bottom CrSBr monolayers, respectively. We apply h along the hard axis of the bottom monolayer (α_1 = 90°), and α_2 = 35° for the top monolayer due to the 55° twist angle (θ_{twist}). Because the two monolayers are decoupled, if dually sweeping h between -0.9 to 0.9 the magnetization process in the bottom monolayer would always evolve along one of the curves in Supplementary Fig. 18d, but the magnetization process in the top monolayer

would do along the double curves of Supplementary Fig. 18b. Then, we can calculate the relative angle ($\theta = \beta_1 - \beta_2$) of the two magnetizations. Supplementary Fig. 19b shows the result of the green curve in Supplementary Fig. 18d being adopted, and Supplementary Fig. 19c shows the results if the blue curve is adopted. It is seen that when the magnetization flips to its time-reversal copy in the pinned layer, the shape of θ–h curve is mirrored. Both results indicate θ has two values of θ_{twist} and π - θ_{twist} at ZF. Because tunneling conductance (G) is related to the relative angle (θ), see Eq. (8) of the main text, we obtain bistable tunneling conductances at ZF.

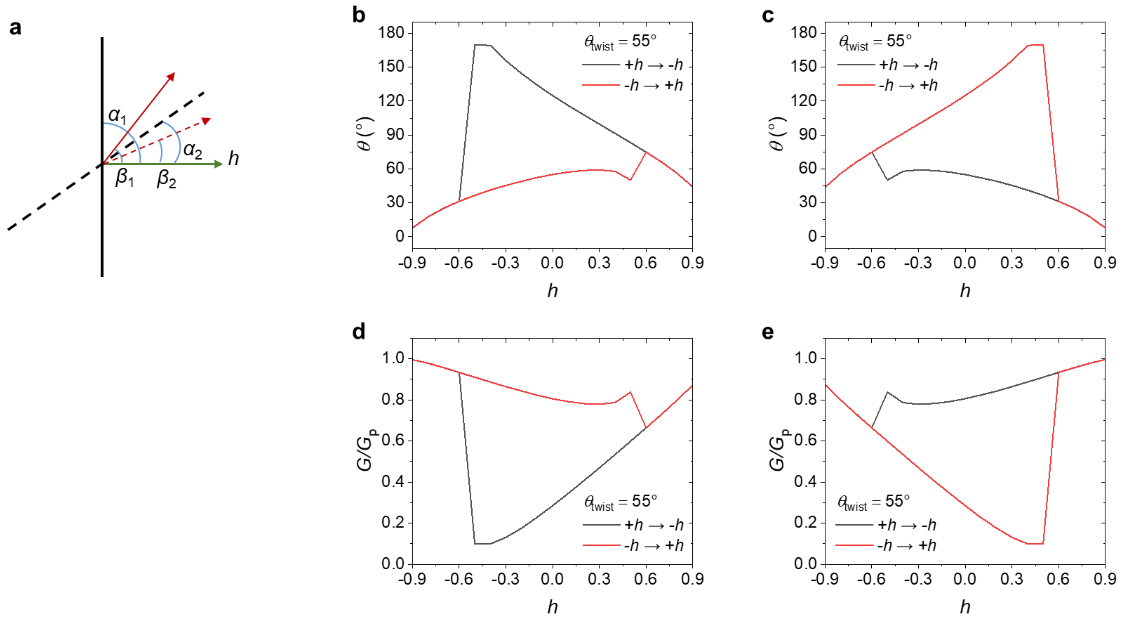

Supplementary Fig. 19 | The evolution of tunneling conductance with an external magnetic field sweeping. a, A schematic diagram of two magnetizations (solid and dashed red arrows) in an external magnetic field. Black lines are the easy axes. **b**-**c**, Relative angle ($\theta = \beta_1 - \beta_2$) of the two magnetizations in a dual sweeping external magnetic field. **d**-**e**, Tunneling conductance in a dual sweeping external magnetic field.

Furthermore, using Eq. (8) of the main text and simply considering $T_{\mathrm{AP}}(\theta) = \frac{1}{11} T_{\mathrm{P}}(\theta)$ for all θ, $G \propto T(\theta)$ as a function of h can be quantitatively calculated; that is, we get $\frac{G}{G_{\mathrm{P}}} = \frac{6}{11} + \frac{5}{11}\cos\theta$ by substituting $T_{\mathrm{AP}}(\theta) = \frac{1}{11} T_{\mathrm{P}}(\theta)$ into Eq. (8) of the main text and then the corresponding results of Supplementary Fig. 19b and c are obtained in Supplementary Fig. 19d and e, respectively. The final results well reproduce the experimental results of Supplementary Fig. 15e and Extended Data Fig. 6d at Φ = 210°. Including ZF NV and mirror symmetry, the sloped relationship between tunnel currents and the external field is also captured.

a

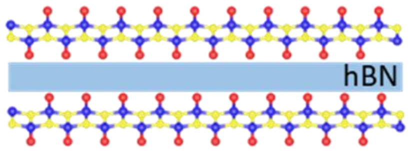

b

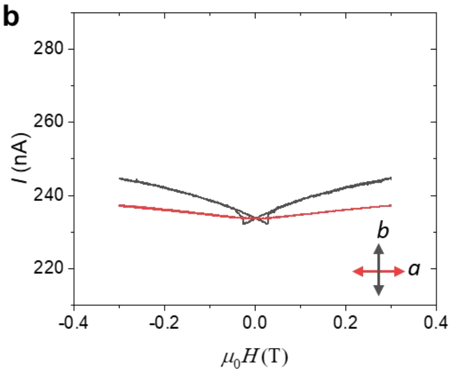

Supplementary Fig. 20 | No ZF NV in an untwisted CrSBr monolayer/hBN monolayer/CrSBr monolayer. a, Schematic of the MTJ. **b**, Tunneling current *versus* field at 2 K with field oriented along the directions indicated by the inset. The nonoverlap between the forward-sweeping and backward-sweeping along *b-axis* could originate from the magnetic domain in the junction area.

To realize ZF NV, the key is to make the magnetization in one CrSBr flake of the twisted structure always evolve along a curve when backward and forward sweeping field and only one direction is reached at ZF, which means this magnetization is pinned. In contrast, the magnetization in the other CrSBr flake can reach two directions at ZF as a free layer. Because the pinned effect is α-dependent (Supplementary Fig. 14b), we can control the sweeping range of the external field to make one flake pinned and the other one free (Extended Data Fig. 3f,g). Note that the pinning correlated with a twist has been discussed in Supplementary Note 1, which is necessary to make an MTJ functional. In an untwisted CrSBr monolayer/hBN monolayer/CrSBr monolayer MTJ (Supplementary Fig. 20), we do not observe any ZF NV due to the absence of pinning even if the inserted hBN layer decouples the interface.

1. Parkin, S.S. et al. Giant tunnelling magnetoresistance at room temperature with MgO (100) tunnel barriers. *Nat Mater* **3**, 862-867 (2004).
2. Gallagher, W.J. & Parkin, S.S.P. Development of the magnetic tunnel junction MRAM at IBM: From first junctions to a 16-Mb MRAM demonstrator chip. *IBM Journal of Research and Development* **50**, 5-23 (2006).
3. Boix-Constant, C. et al. Multistep magnetization switching in orthogonally twisted ferromagnetic monolayers. *Nat Mater* **23**, 212-218 (2024).
